# Do paediatric early warning scores relate to emergency department outcomes for children aged 0–2 years brought in by ambulance?

**DOI:** 10.29045/14784726.2019.03.3.4.8

**Published:** 2019-03-01

**Authors:** William M. Broughton, Ian K. Maconochie

**Affiliations:** University of Hertfordshire; Imperial College Healthcare NHS Trust

**Keywords:** ambulance, early warning score, paediatrics, PEWS

## Abstract

**Introduction:**

Ambulance service policy requires paramedics in certain parts of the UK to transport children aged 0–2 years to hospital, regardless of their presenting complaint. While there are a number of paediatric early warning scores (PEWS) that exist to detect deterioration in the hospitalised child, no study has considered the potential relationship between a PEWS recorded by the ambulance service and emergency department (ED) outcome. This study aims to evaluate and understand the potential utility of PEWS in an ambulance service setting.

**Methods:**

A retrospective analysis of patient reports was undertaken, using data from the London Ambulance Service (LAS) and St Mary’s Hospital, Paddington, collected over a 12-month period (June 2013 to June 2014). PEWS were calculated using LAS vital signs and compared against ED discharge outcomes.

**Results:**

From a randomised sample of 300 patient records, 169 were included in the final analysis. A total of 100/169 (59.2%) were discharged to home, 30 (17.8%) referred to their GP and 18 (10.7%) were admitted following assessment in the ED. A total of 87/169 had a PEWS of 1, with the vast majority of PEWS 1 (n = 64) resulting in discharge to home. *PEWS for admission* showed low sensitivity (6.8–10.12%) across all scores. Specificity was high for lower scores, but positive predictive values (PPV) were low. *PEWS for GP referral* also demonstrated low sensitivity (15.53–18.12%) but with higher specificity across all scores. PPV was high for scores > 2 and a PEWS of 2. *PEWS for discharge to home* showed higher sensitivity and specificity than other outcomes, with a PEWS of 2 demonstrating high sensitivity (61.07%), specificity (55.0%) and the PPV was 90%.

**Conclusion:**

PEWS demonstrated high specificity, but poor sensitivity in all outcome measures. As a potential diagnostic test to predict ED outcome, in this study PEWS performed poorly. Further work is required to determine the utility of PEWS, or other early warning scores, for use in an out-of-hospital setting.

## Introduction

Ambulance calls involving children only account for approximately 10% of all calls, and of those, only 5% require resuscitation or advanced intervention (Jewkes, 2001). In 2009, the Royal College of Paediatrics and Child Health (RCPCH) wrote to all UK ambulance services advocating that all children under 2 years of age should be taken to hospital, regardless of their complaint. This guidance was subsequently incorporated into London Ambulance Service’s (LAS) paediatric care policy (London Ambulance Service NHS Trust, 2012).

While this means that a child aged 2–5 years can be managed at home, within LAS NHS Trust the ambulance clinician must complete a direct referral to another suitable healthcare professional (London Ambulance Service NHS Trust, 2012). This could be in the form of a GP appointment or out-of-hours visit, or the child may be conveyed to an alternative care pathway, such as an NHS walk-in-centre. One might consider this recommendation to be a safe one, considering the findings of Houston and Pearson (2010) regarding the lack of standardised education and training for ambulance clinicians. However, there exist no published data from a UK ambulance service regarding paediatric conveyance by ambulance services, and therefore no data to inform this decision.

A number of paediatric early warning score (PEWS) systems have been developed following the validation of the Brighton score in 2005 (Monaghan, 2005), but are all for use within a hospital setting. The scoring systems use physiological data such as heart rate and respiratory rate to generate a score, which then prompts clinical staff to carry out a particular action, for example requesting a medical review of the patient. Implementation of such scoring systems is widespread across hospital inpatient settings, but studies have yet to reliably validate the various scoring tools (Chapman, Grocott, & Franck, 2010; Winberg, Nilsson, & Aneman, 2008). In adults, NEWS2 is now a commonly used tool across the UK health system, and work is ongoing to develop a similar national scoring system for paediatric patients (NHS England, personal communication, 9 April 2018; Royal College of Paediatrics and Child Health, personal communication, 2 June 2018; Royal College of Physicians, 2017).

This study aims to determine how accurately PEWS, calculated from ambulance service data, can predict emergency department (ED) outcome.

## Methods

A retrospective analysis of routine data collected by the LAS NHS Trust and the ED at St Mary’s Hospital, Paddington (Imperial College NHS Trust) was undertaken. The data included illness/injury codes, vital signs, triage information and ED outcome/discharge information.

All paediatric patients aged 0–2 years, who were attended by LAS and conveyed to St Mary’s ED in the period from June 2013 to June 2014, were included if they did not meet any of the following exclusion criteria:

- Patients conveyed under blue light conditions (deemed critically unwell by LAS), including patients conveyed under major trauma protocol.
  - Excluded as these children were likely to be admitted and may not have had a full set of vital signs recorded (dependent on severity of illness/injury).
- Inter-hospital transfers undertaken by LAS or approved LAS contractor.
- Incomplete LAS data (PEWS could not be calculated).
- Unable to match LAS and St Mary’s data, or missing data.

Data extracted from LAS report forms were entered into a spreadsheet and a retrospective PEWS calculated using charts produced by the NHS Institute for Innovation and Improvement (Supplementary 1 and 2). In addition, the relevant hospital record outcome and discharge data were also entered to enable comparison between ambulance service and hospital data. Where data were missing from hospital records, the patients were excluded, as the researcher (lead author) was not permitted to access hospital notes to locate missing information.

The PEWS system used for this study allocates a score of ‘1’ for parental concern. As each patient was being conveyed to hospital by emergency ambulance following a 999 call or referral from the NHS 111 service, it was assumed that parental concern existed for every patient.

A power calculation was performed to determine the required sample size with a 5% margin of error, that is the accepted chance of error to account for any miscalculation. Using a population of 1162 and a confidence interval of 95%, the recommended sample size was 289.

Descriptive statistics were analysed and produced using Microsoft Excel for Mac 2011. Sensitivity and specificity were calculated using data collected in Excel and then transferred into IBM SPSS Statistics 22.0 to determine the sensitivity, specificity, positive predictive value (PPV), negative predictive value (NPV) and 95% confidence intervals.

Patient identifiable data were held in a secure folder on the LAS computer network and could only be accessed by the lead author on a LAS terminal. No patient identifiable data were removed from the NHS site, nor were they stored on any portable media.

### Ethics

A favourable opinion was obtained from the Stanmore Research Ethics Committee. The study was also submitted for site specific assessment at each participating NHS Trust. NHS Trust research approval was obtained from the Joint Research Compliance Office at Imperial College London as well as the Clinical Audit and Research Unit at LAS NHS Trust, before accepting participants into the study.

Since the study utilised fully anonymised and routinely collected data, consent was not required and patients were not informed of their inclusion in this study.

## Results

**Figure 1:**
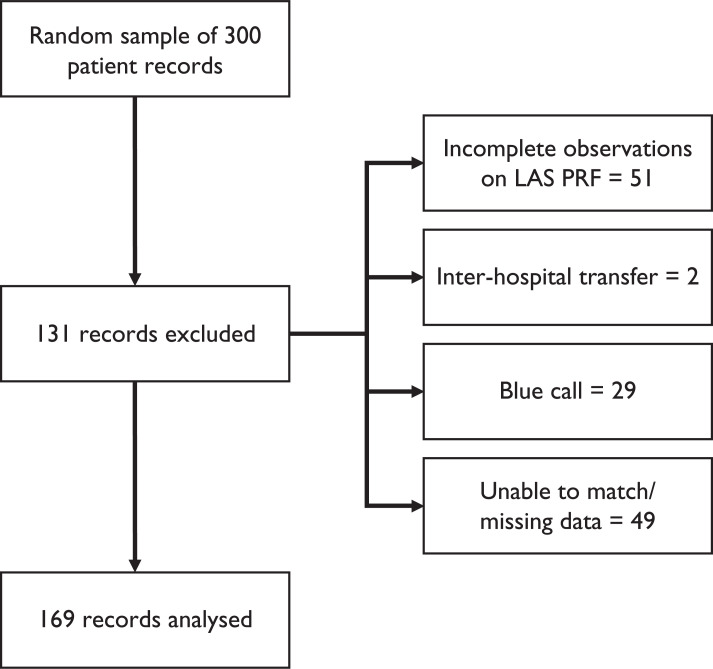
Figure 1. Enrolment diagram.

There were 1162 ambulance attendances to the paediatric emergency department (PED) at St Mary’s Hospital (Paddington) during the study period. A random sample of 300 patient records was included for analysis in this project and the sample was taken prior to any data matching. Following initial data collection, a total of 169 records were found to be complete and suitable for inclusion, and 131 records were excluded from the analysis (Figure 1).

**Figure 2:**
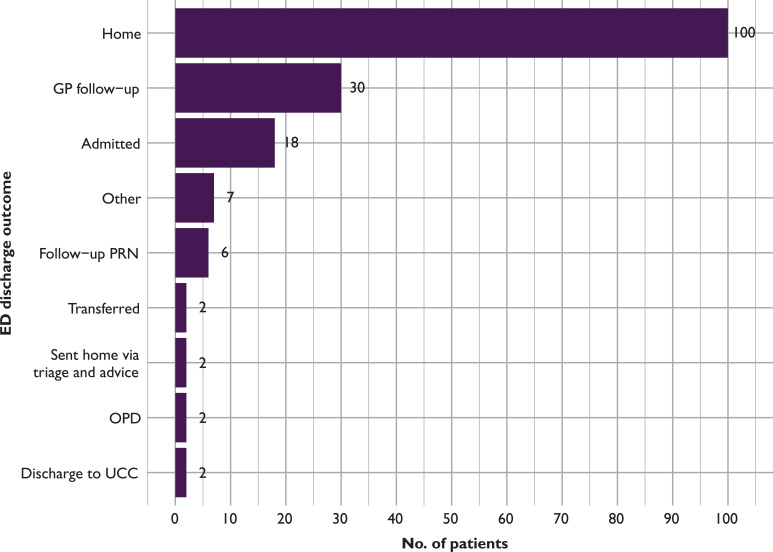
Figure 2. Paediatric emergency department outcome.

Note: Other = social worker, medical OPD, health visitor, did not wait for doctor/ENP, did not complete treatment, awaiting speciality review, and A&E review clinic.

Of the 169 patients with PED outcome data, 18/169 (10.7%) patients were admitted and 30/169 (17.8%) were discharged for follow-up by the patient’s own GP. The majority, a total of 100 (59.2%) patients, were discharged home from the PED following assessment and/or treatment. The remaining 21 (12.4%) patients had 11 different ED outcomes (Figure 2).

Table 1 demonstrates patient outcome stratified by PEWS (for most common emergency department outcome). Overall, PEWS performed poorly as a tool for prediction across all ED discharge outcomes.

**Table 1.** Patient outcome stratified by PEWS (for most common emergency department outcome).

Outcome	PEWS			
	1	2	3	4 or more
Admission	7	6	3	2
GP referral	16	11	2	0
Discharged home	64	27	9	0
Total	87	44	14	2

### PEWS and admission

Table 2a summarises the data for ambulance service PEWS with respect to hospital admission. With increasing PEWS, sensitivity rose, however specificity decreased. A PEWS of 1 showed the highest specificity for admission (83.3%), but the lowest sensitivity (6.8%). For patients admitted from the ED, PEWS demonstrated very low sensitivity, indicating that it is highly unlikely that patients with a PEWS of 1 would require admission (true negatives), but with sensitivity of 6.8% the test is very poor at identifying those who do need admission within this cohort.

**Table 2.** Ambulance PEWS sensitivity, specificity and positive and negative predictive value with respect to emergency department outcome.

PEWS	Sensitivity % (95% CI)	Specificity % (95% CI)	PPV % (95% CI)	NPV % (95% CI)
**a) ED outcome: admission**				
1	**6.80**	**83.33**	**38.89**	**36.42**
	2.78–13.50	72.13–91.38	17.30–64.25	28.75–44.64
2	**8.72**	**75.00**	**72.20**	**9.93**
	4.73–14.46	50.9–91.34	46.52–90.31	5.67–15.50
3	**9.64**	**33.30**	**88.89**	**0.66**
	5.61–15.18	0.84–90.57	65.29–98.62	0.02–3.63
≥ 4	**10.12**	**0.00**	**94**.**44**	**0.00**
	6.01–15.71	0.00–97.50	72.71–99.86	0.00–2.41
**b) ED outcome: GP referral**				
1	**15.53**	**78.79**	**53.33**	**37.42**
	9.15–24.00	66.98–87.89	34.33–71.66	29.36–46.01
2	**18.12**	**85.00**	**90.00**	**12.23**
	12.29–25.26	62.11–96.79	73.47–97.89	7.29–18.86
3	**17.47**	**66.67**	**96.67**	**1.44**
	12.02–24.12	9.43–99.16	82.78–99.92	0.17–5.10
≥ 4	**17.86**	**100.00**	**100.00**	**0.72**
	12.38–24.50	2.50–100.00	88.42–100.00	0.02–3.94
**c) ED outcome: discharged home**				
1	**62.14**	**45.45**	**64.0**	**43.48**
	52.04–71.51	33.14–58.19	53.79–73.36	31.58–55.96
2	**61.07**	**55.0**	**91.0**	**15.94**
	52.75–68.95	31.53–76.94	83.60–95.80	8.24–26.74
	52.75–68.95	31.53–76.94	83.60–95.80	8.24–26.74
	52.75–68.95	31.53–76.94	83.60–95.80	8.24–26.74
	52.75–68.95	31.53–76.94	83.60–95.80	8.24–26.74
3	**60.24**	**100.0**	**100.0**	**4.35**
	52.37–67.74	29.24–100.00	96.38–100.00	0.91–12.18
≥ 4	**59.52**	**100.0**	**100.0**	**1.45**
	51.69–67.02	2.50–100.00	96.38–100.00	0.04–7.81

NPV: negative predictive value; PPV: positive predictive value.

### PEWS and GP referral

Table 2b summarises the data for ambulance service PEWS with respect to GP referral. Sensitivity was low across all PEWS, however specificity increased with increasing PEWS. A PEWS of ≥ 4 showed the highest specificity for admission (100.0%), but low sensitivity (17.86%). For patients referred to the GP from the ED, PEWS demonstrated low sensitivity ranging from 15.53% (for PEWS 1) to 17.86% (for PEWS ≥ 4). Despite high specificity, a low sensitivity means that PEWS demonstrates no diagnostic accuracy in detecting patients suitable for referral to their GP in this cohort of patients.

### PEWS and discharge to home

Table 2c summarises the data for ambulance service PEWS for patients discharged home from the ED. Sensitivity was low across all PEWS, however specificity increased with increasing PEWS. A PEWS of ≥ 3 showed the highest specificity for discharge home (100.0%), but lower sensitivity (60.24% and 59.52%). For patients discharged to home from the ED, PEWS demonstrated higher sensitivity than the previous outcome measures, ranging from 59.52% (for PEWS ≥ 4) to 62.14% (for PEWS 1). Specificity was highest for a PEWS of ≥ 4 at 100%, but this had a wide 95% CI (2.50%–100.0%) as well as a low NPV. A PEWS of ≥ 3 is a poor diagnostic test for home discharge.

One unintended finding from this study was the number of LAS patient report forms (PRFs) missing basic vital signs information. A clinical audit is required to determine the scale of this problem, followed by a more detailed study to understand the reasons for missing data on ambulance report forms. Previously published work has identified some issues around paramedic education with regard to paediatric patients, and this may be a contributing factor for why vital signs information was missing in this study (Houston & Pearson, 2010; Jewkes, 2001). In 2017, the Royal College of Nursing published a set of ‘Standards for assessing, measuring and monitoring vital signs in infants, children and young people’ (Royal College of Nursing, 2017) and this variation in ambulance reporting of vital signs would suggest the need for a similar guidance to be issued to ambulance staff.

## Discussion

Increasing pressure on EDs to manage patients within the 4-hour target led to PEWS being explored as a means to triage and predict admission (Bradman & Maconochie, 2008; Bradman, Borland, & Pascoe, 2014; Gold, Mihalov, & Cohen, 2014; Lillitos, Hadley, & Maconochie, 2016; Seiger, Maconochie, Oostenbrink, & Moll, 2013). Recent work by Lillitos et al. (2016) found that, while a high score should be taken seriously, low scores are inadequate to rule out the need for admission and to detect serious illness. Bradman and Maconochie (2008) focused on the triage application of PEWS, and found it to be of limited value in predicting admission, owing to the population arriving with undifferentiated presenting complaints. In their study, low PEWS demonstrated high specificity, that is a patient scoring < 2 was unlikely to need admission (Bradman & Maconochie, 2008). If a low PEWS was also able to demonstrate high sensitivity, thus identifying those patients who do need admission, the results could be more valid. However, with this high specificity the many false positive results only serve to drown out the true positives.

Keogh (2014) has suggested that more patients need to be treated in the community, but with Jewkes, Houston and Pearson suggesting that paediatric training and education for ambulance staff is lacking, we must consider clinical practice more carefully to ensure that patients can be safely managed by trained ambulance clinicians (Houston & Pearson, 2010; Jewkes, 2001).

There is currently a limited body of evidence to inform the decision to transport or treat on scene with regard to the paediatric population. Kost and Arruda (1999) examined a US system (Medicaid) to assess the appropriateness of ambulance transportation to a paediatric ED. From the sample of 294 patients, ranging from 2 weeks to 19 years of age, 28% (n = 82) of patients were deemed to have been brought in by ambulance unnecessarily. The criteria for appropriate ambulance use were determined by seven paediatric emergency medicine physicians, and included cardiac arrest, respiratory distress and motor vehicle collision. What the paper does not attempt to analyse is those patients for whom ambulance use was deemed appropriate, to establish their ED outcome in order to validate the list of criteria for appropriate ambulance use. It is also difficult to extrapolate these data to a UK system, as the American model of insurance and paid-for health services is a major factor in this paper. Camasso-Richardson, Wilde, and Petrack (1997) also examined the US system, and in a sample of 92 patients found that 62% were transported by ambulance unnecessarily. They collected follow-up data for 85/92 patients and discovered that many used the ambulance system because they had no other means of transportation; 78% (68/92) were able to return home without any assistance. A comprehensive search of UK literature revealed no published UK or UK-comparable data on the subject of paediatric non-conveyance or admission reduction by ambulance staff.

Overall, PEWS has been shown to have some degree of high specificity in relation to all outcome measures, but often with wide confidence intervals. PEWS is poorly sensitive across all outcomes. The implications for clinical practice are that if PEWS is to be used by ambulance staff, a high score should warrant concern for the presenting child (Monaghan, 2005; Newton, Tunn, Moses, Ratcliffe, & Mackway-Jones, 2014). However, a low score should not be taken to suggest an increased likelihood of non-admission. Lower scores still resulted in admission. A low score should thus be regarded as indicating a potentially ‘well’ child, but must not negate the need for thorough clinical assessment and reasoned clinical decision-making by ambulance staff (Newton et al., 2014).

A recommendation of this study will be to undertake an audit of current practice, to establish the incidence of incomplete data entry for paediatric patients and to determine the reason behind this lack of compliance from clinical staff.

Future work should include a larger multi-centre study, taking place over at least a 12-month period. A larger study of this kind would reduce the population bias identified in this article, and a larger sample will allow for better statistical analysis of PEWS performance in relation to ED outcomes, particularly for PEWS with scores greater than 3. A further study should also consider examining ED diagnosis data and comparing that with the clinical impressions recorded by ambulance staff, in order to ascertain the accuracy of diagnosis during ambulance assessment. The clinical utility of a scoring system is yet to be determined, but in order to design such a system, accuracy of diagnosis alongside PEWS should be evaluated.

### Technical aspects and limitations

This study was a retrospective analysis of patient data collected over a 12-month period from June 2013 to June 2014. The type of data that were required for this study is routinely recorded by both the ambulance service and ED, meaning a significant amount of time was saved in comparison to prospective data collection. As part of the project approval conditions, the researcher (lead author) was not permitted to access identifiable patient information from Imperial College Healthcare NHS Trust (ICH), and so it was not possible to use other determinants, such as ‘date of birth’, to match the records.

A second limitation of the data was found when analysing the LAS PRF, where 51 patients were excluded from the dataset due to vital signs being missing from the report form. An unintended outcome of the project was identifying that, from a small sample of 300 patients, 17% of records were missing basic vital signs information. The LAS Paediatric Care Policy (London Ambulance Service NHS Trust, 2012) requires clinical staff to obtain at least two full sets of observations, taken 20 minutes apart, including:

- respiratory rate;
- heart rate;
- capillary refill time;
- capillary blood glucose;
- pulse oximetry (where equipment is available);
- temperature.

Complete data were obtained for 169 patients using the methodology described above, which fell short of the target sample of 300 needed for a 95% level of confidence. In terms of data acquisition, the limitation of not being able to access patient information (such as date of birth) from ICH and the incompleteness of LAS PRFs meant that the principal investigator (PI) was unable to collect sufficient data to achieve a sample size of 300 for analysis. As the project was limited by time due to requirements for an MSc thesis, the researcher (lead author) was unable to resample the data to build an adequate sample size. Access to the LAS PRF system was granted for a specific amount of time, as part of the project approval process. These are important barriers for future research in this area.

In this study, a minimum PEWS of 1 was automatically assigned for each patient based on assumed parental concern, due to the parents having called 999 to request an emergency ambulance. It is recognised, however, that this assumed concern might not always be present if ambulance staff have been able to provide early reassurance on arrival at the scene. However, as the patients examined for this study were also conveyed to an ED, one might reasonably assume concern on the part of the ambulance staff as well. Therefore, in the context of emergency ambulance calls, an automatic score of 1 for concern in this study is justifiable.

## Conclusion

This is the first study to date examining the relationship between PEWS and ED discharge outcome for patients brought in by the ambulance service. PEWS demonstrated high specificity, but poor sensitivity in all outcome measures. As a potential diagnostic test to predict ED outcome, in this study PEWS performed poorly. Further work is required to understand the variation in vital sign documentation by ambulance staff and to determine the utility of PEWS, or other early warning scores, for use in an out-of-hospital setting.

## Acknowledgements

The authors thank Adam Smith for providing an anonymised dataset from Imperial College Healthcare.

## Author contributions

WMB devised the original protocol, carried out data collection and analysis and prepared the manuscript. IKM advised on original protocol and methodology, supported the data analysis and reviewed the manuscript.

## Conflict of interest

None declared.

## Ethics

Internal R&D approval from Imperial College, Imperial College Healthcare NHS Trust and London Ambulance Service NHS Trust. A favourable opinion was obtained from the local NHS REC.

## Funding

None.
